# Boosted Efficiency of Fe_2_O_3_ for Photocatalytic CO_2_ Reduction via Engineering Fe−O−Ti Bonding

**DOI:** 10.1002/advs.202409002

**Published:** 2024-11-29

**Authors:** Jingyi Wu, Wei Wang, Xudan Chen, Qiquan Luo, Changzeng Yan, Zhen Jiao, Yuehui Li

**Affiliations:** ^1^ College of Smart Energy Shanghai Jiao Tong University Shanghai 200240 P. R. China; ^2^ Lanzhou Institute of Chemical Physics (LICP) Chinese Academy of Sciences Lanzhou 730000 P. R. China; ^3^ School of Chemistry and Chemical Engineering Jiangsu Key Laboratory for Biomaterials and Devices Southeast University Nanjing 211189 P. R. China; ^4^ School of Chemistry and Chemical Engineering Ningxia University Yinchuan 750021 P. R. China; ^5^ Institutes of Physical Science and Information Technology Anhui University Hefei 230601 China; ^6^ Carbon‐Negative Synthetic Biology for Biomaterial Production from CO2 (CNSB) Campus for Research Excellence and Technological Enterprise (CREATE) 1 CREATE Way Singapore 138602 Singapore

**Keywords:** CO_2_ photoreduction, electrostatic self‐assembly, Fe_2_O_3_, KH‐550, Ti_3_C_2_ MXene

## Abstract

Visible light‐driven photocatalytic CO_2_ reduction (CO_2_RR) offers a sustainable and promising solution to environmental and energy challenges. However, the design of efficient photocatalysts is hindered by poor interface interactions in heterojunctions and a limited understanding of reaction kinetics. A modified Fe_2_O_3_ photocatalyst, M‐Fe_2_O_3_@MXene, is introduced featuring KH‐550‐modified M‐Fe_2_O_3_ hollow nanocubes coated with MXene, constructed via an electrostatic and Fe−O−Ti bonding self‐assembly method. This design achieves an unprecedented CO production rate of 240 µmol g⁻¹ h⁻¹ among non‐noble metal catalysts (8.6 folds vs Fe_2_O_3_). The Fe−O−Ti sites enhance *COOH intermediate formation and CO production through higher electron deficiency of Fe^3+^ and rapid charge transfer. This study offers new insights on the use of functional metal oxides and high‐quality Mxene layers to design efficient metal oxide‐based photocatalysts.

## Graphical abstract

Single‐layer MXene‐coated hollow Fe_2_O_3_ nanocube catalyst (M‐Fe_2_O_3_@MXene) is fabricated via an electrostatic and Fe−O−Ti bonding self‐assembly method. Experiments and theoretical calculations imply that interfacial Fe−O−Ti bonding increases the photocatalytic CO_2_ reduction (p‐CO_2_RR) activity by accelerating charge transfer and separation and regulating electronic property of Fe sites. This catalyst outperforms previous reports using Fe‐based photocatalysts with a CO production rate of 240 µmol g^−1^ h^−1^.

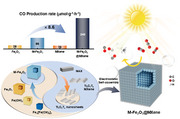

## 1 Introduction

Developing more efficient catalysts for the conversion of CO_2_ into value‐added chemicals is critical for approaching the goal of zero carbon.^[^ ^1^ ^]^ Photocatalytic CO_2_RR harnesses solar energy and offers a potential blueprint.^[^ ^2^ ^]^ Single‐metal oxides account for a considerable part of photocatalysts, such as TiO_2_,^[^ ^3^ ^]^ ZnO,^[^ ^4^ ^]^ SnO_2_,^[^ ^5^ ^]^ etc. Compared to these semiconductors, iron oxide (Fe_2_O_3_) is also considered a competitive candidate due to its low cost, high abundance, and wide bandgap (2.2 eV).^[^ ^6^ ^]^ Yet challenges remain in low selectivity and activity, mainly due to the low conduction band (CB) bottom level to provide high‐energy photogenerated electrons.^[^ ^7^ ^]^ Specifically, CO_2_ conversion using FeO*_x_*‐based nanoparticles usually favors generation of H_2_.^[^ ^8^ ^]^ Wang et al.^[^ ^9^ ^]^ constructed an Al−O bridged g‐C_3_N_4_/α‐Fe_2_O_3_ Z‐scheme heterojunctions to promote charge transfer and separation and achieved a CO production rate of 24.0 µmol g^−1^ h^−1^. Zhao et al.^[^ ^10^ ^]^ reported a FeO*_x_*/ZSM‐5 heterojunction that exhibits a rate of 17.6 µmol g^−1^ h^−1^ for CO by furnishing favorable band gaps and active sites for CO_2_ adsorption/activation. Nevertheless, utilizing iron oxides as efficient photocatalysts in photo‐CO_2_RR remains a challenge respecting theoretical understanding and catalyst design.^[^ ^11^ ^]^ And, literature surveys reveal that the photo‐CO_2_RR activity of FeO*_x_*‐based materials is unsatisfying since their production rate of CO remains at a single or two‐digit level µmol g^−1^ h^−1^ (**Table** **1**).

**Table 1:** Comparison of photocatalytic CO_2_ reduction performance for iron oxide‐based and Ti_3_C_2_‐based photocatalysts in previous literature and this work.

Photocatalysts	Synthesis	Major products	Production rate [µm g^−1^ h^−1^]	Refs.
g‐C_3_N_4_/α‐Fe_2_O_3_	Hydrothermal method	CH_3_OH	5.6	[9]
α‐Fe_2_O_3_/BCN	One‐pot ionothermal method	CO	55.1	[24]
FeO*_x_*/ZSM‐5	Function ion preadsorption method	CO	17.6	[10]
α‐Fe_2_O_3_/Cu_2_O	Hydrothermal‐deposition	CO	5.0	[25]
Co_3_O_4_@CoFe_2_O_4_	Solvothermal reaction	CO	66.2	[26]
Ti_3_C_2_/Bi_2_WO_6_	In situ growth method	CH_4_	1.8	[21]
TiO_2_/C_3_N_4_/Ti_3_C_2_	In situ growth method	CO	4.4	[20a]
Ti_3_C_2_ QDs/Cu_2_O NWs/Cu	Self‐assembly strategy	CH_3_OH	78.5	[14a]
Pt‐TiO_2_NW/Ti_3_C_2_	Oxidation reactions	CO/CH_4_	3.8/3.6	[22]
Cs_2_AgBiBr_6_/MXene	Self‐assembly strategy	CO	50.6	[23]
Fe_2_O_3_	Hydrothermal method	CO	28	This work
M‐Fe_2_O_3_	Surface modification	CO	86	This work
Ti_3_C_2_ MXene	LiF/HCl etching	CO	23	This work
M‐Fe_2_O_3_@MXene	Electrostatic self‐assembly	CO	240	This work

2D materials have attracted broad attention due to their unique physical, chemical, and electronic properties.^[^ ^12^ ^]^ As a state‐of‐the‐art 2D material, MXene offers promising applications in various fields (energy storage and conversion,^[^ ^13^ ^]^ catalysts,^[^ ^14^ ^]^ sensors,^[^ ^15^ ^]^ water treatment,^[^ ^16^ ^]^ and so on^[^ ^17^ ^]^) due to its large specific surface area, and excellent metallic conductivity.^[^ ^18^ ^]^ MXene‐based composite material provides abundant active sites and accelerated ion transfer with flexibility.^[^ ^19^ ^]^ Existing research recognizes the critical role played by MXene in CO_2_RR, which is capable of promoting CO_2_ adsorption and activation, improving the photothermal effect of metal nanoparticles, activating the photothermal CO_2_RR, and facilitating the separation of photocarriers.^[^ ^20^ ^]^ Cao et al.^[^ ^21^ ^]^ prepared a novel 2D/2D heterojunction of ultrathin MXene/Bi_2_WO_6_ nanosheets by in situ growth method and provided a protocol for constructing MXene coupled nanostructures for CO_2_RR. The product yield with optimized mass ratios of Ti_3_C_2_ to Bi_2_WO_6_ is 1.8 µmol g^−1^ h^−1^ for CH_4_ and 0.4 µmol g^−1^ h^−1^ for CH_3_OH, respectively. Lee et al.^[^ ^22^ ^]^ demonstrated the enhanced activity of CO_2_ reduction to CO (3.8 µmol g^−1^ h^−1^) and CH_4_ (3.6 µmol g^−1^ h^−1^) by using platinum quantum dots‐decorated MXene‐driven TiO_2_ nanowire to form Pt−O bonds serving as active sites. A self‐assembly Cs_2_AgBiBr_6_/Ti_3_C_2_ lead‐free perovskite‐MXene heterostructure with excellent charge separation was developed by Xing et al., achieving a high electron consumption yield of 50.6 µmol g^−1^ h^−1^.^[^ ^23^ ^]^ So far, the catalytic activity and charge transfer ability of MXene‐based photocatalysts need to be further improved.

With precise interface engineering, enhanced intrinsic activity and charge transfer ability of Fe_2_O_3_ might be achieved with the assistance of MXene. From the point of view of electronic properties, iron oxides with O_v_ serve as Lewis acid sites, and the MXene could serve as Lewis base due to its abundant surface groups (─OH, ─F). Besides, an interfacial chemical bond might not only provide the intense driving force for photoelectron transfer but also increase intrinsic activity for CO production. Therefore, we constructed a noble‐metal‐free catalyst by combining the modified Fe_2_O_3_ nanocubes wrapped by single‐layer MXene featuring Fe−O−Ti bonding to achieve improved performance of photo‐CO_2_RR catalysis. With MXene membrane coating, the photocatalytic CO production rate (240 µmol g^−1^ h^−1^) of as‐synthesized M‐Fe_2_O_3_@MXene exhibited 8.6‐fold compared to Fe_2_O_3_ microcubes. X‐ray photoelectron spectroscopy (XPS) and DFT calculation further confirmed that abundant active interfacial Fe−O−Ti sites substantially lower the Gibbs free energy barrier for CO_2_ reduction to CO on iron(III) oxide sites via enhanced charge transfer and separation processes. It is also proposed that the Fe−O−Ti bonding effect increased the intrinsic activity of Fe sites.

## 2 Results and Discussion

**Scheme 1:**
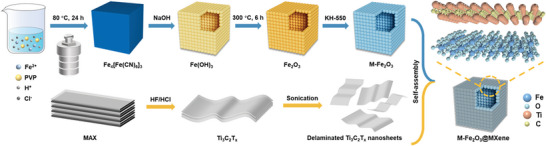
Schematic illustration of synthesizing M‐Fe_2_O_3_@MXene.

To expose a high concentration of Fe─O sites for bonding and guarantee excellent interaction between Fe_2_O_3_ and MXene, a hollow nanocube structure of Fe_2_O_3_ was chosen. **Scheme** **1** illustrates the preparation process of the self‐assembly M‐Fe_2_O_3_@MXene. The highly uniform M‐Fe_2_O_3_ nanocubes were prepared through a hydrothermal method and subsequential surface modification. First, Fe_4_[Fe(CN)_6_]_3_ solid nanocubes were prepared by hydrothermal treatment of the Fe^2+^ precursor and PVP acid solution. Subsequently, Fe(OH)_3_ was obtained by etching the template with NaOH to form a highly uniform hollow structure. Then, Fe_2_O_3_ was prepared by pyrolyzing the hydroxide. Specifically, after heat treatment, (3‐aminopropyl) triethoxysilane (KH‐550) with abundant NH_2_ group was used for surface modification to form M‐Fe_2_O_3_. MXene single layers were prepared using HF etching methods. Ti_3_C_2_T*_x_* MXene was obtained through etching MAX (Ti_3_AlC_2_) in the LiF and HCl mixture solution. Then, the accordion‐like MXenes were delaminated into single layers by careful ultrasound treatment and separation. Owing to the surface functional groups of MXene nanosheets (T*_x_*, i.e., −F, −OH, and −O) and modified Fe_2_O_3_ nanocubes (−OH and −NH_2_), two materials were spontaneously self‐assembled, with a thin layer of flexible MXene film coating on M‐Fe_2_O_3_ hollow nanocubes.^[^ ^27^ ^]^

**Figure 1:**
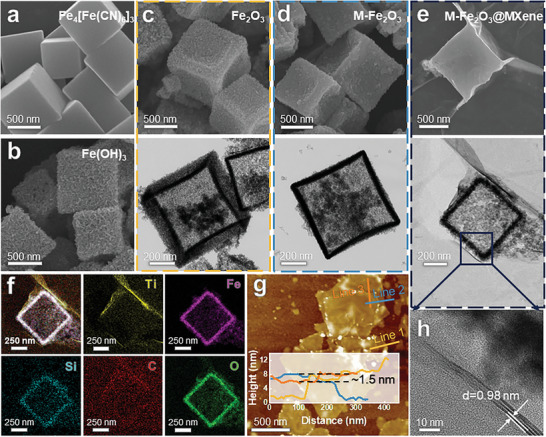
SEM and TEM images of a) Fe_4_[Fe(CN)_6_]_3_, b) Fe(OH)_3_, c) Fe_2_O_3_, d) M‐Fe_2_O_3_, e) M‐Fe_2_O_3_@MXene. f) Energy dispersive spectroscopy (EDS) mapping of Ti, Fe, Si, C, and O for M‐Fe_2_O_3_@MXene. g) AFM image of MXene and h) HRTEM images of the contact area of M‐Fe_2_O_3_@MXene.

The morphological features of the resulting materials were observed via scanning electron microscopy (SEM) and transmission electron microscopy (TEM). As shown in **Figure** **1**a–d and Figure S1a–g (Supporting Information), all synthesized Fe‐based materials exhibited a uniform cubic morphology with a side length of ≈600 nm. Furthermore, from Prussian blue (Fe_4_[Fe(CN)_6_]_3_), Fe(OH)_3_, and Fe_2_O_3_ to M‐Fe_2_O_3_, cubic nanostructures were well‐maintained, and a morphological change in‐situ transformation from Fe_4_[Fe(CN)_6_]_3_ solid cubes into specific hollow structures featuring small nanoparticles composed frame was validated by SEM and TEM results. The selected area electron diffraction (SAED) pattern of M‐Fe_2_O_3_ indicated the formation of polycrystalline α‐Fe_2_O_3_ nanocubes made up of small nanoparticles (Figure S1h, Supporting Information). Owing to superiorities such as high specific surface area, enriched interfaces, and small diffusion length, such Fe_2_O_3_ hollow nanostructures usually facilitate chemical reactivity with more exposed Fe−O sites.^[^ ^28^ ^]^ Furthermore, the aggregation of metal oxide nanoparticles caused by their extremely huge surface areas and large surface free energy hinders the homogeneous distribution in water.^[^ ^29^ ^]^ This rational surface modification not only exposes a more effective and stable interfacial area but might also enhance the charge transport of active metal centers in water.^[^ ^30^ ^]^

Besides, ultrathin MXene films were observed to have a clear layered structure (Figure S2a–c, Supporting Information). Atomic force microscopy (AFM) evidenced that Ti_3_C_2_ MXene nanosheets have a uniform thickness of ≈1.5 nm (Figure 1g and Figure S2d, Supporting Information). After electrostatic self‐assembly, a thin layer of MXene is coated on M‐Fe_2_O_3_ nanocubes (Figure 1e). The successful preparation could be proved by energy dispersive spectroscopy (EDS) mapping (Figure 1f), indicating the presence of Fe and O in the cubic framework. At the same time, Ti, F, and C are uniformly distributed in the coating layer. Particularly, high‐resolution TEM (HR‐TEM) (Figure 1h) highlighting the contact area of M‐Fe_2_O_3_ nanocubes and MXene membrane and SAED (Figure S3, Supporting Information) suggested that the coating film was single or few layered, which was in good agreement with the AFM results. This ultrathin and flexible 2D structure allowed for sufficient contact with nanocubes, which was essential for constructing high‐density active sites and enriched Fe−O−Ti bonding.

**Figure 2:**
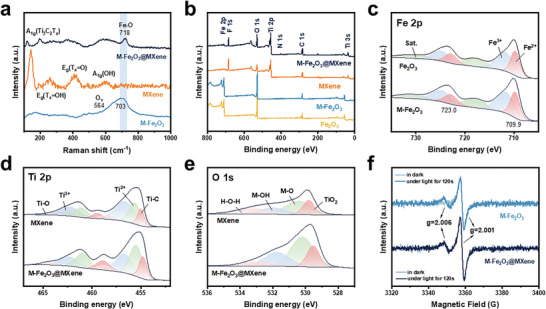
a) Raman spectra and b) XPS survey of Fe_2_O_3_, M‐Fe_2_O_3_, and M‐Fe_2_O_3_@MXene. c) Fe 2p spectra of Fe_2_O_3_ and M‐Fe_2_O_3_. d) Ti 2p. e) O 1s and f) EPR spectra of M‐Fe_2_O_3_ and M‐Fe_2_O_3_@MXene.

As illustrated in Figure S4a (Supporting Information), the X‐ray diffraction (XRD) patterns of the pristine Fe_2_O_3_, M‐Fe_2_O_3_, and M‐Fe_2_O_3_@MXene. The XRD diffraction peaks of Fe_2_O_3_ and M‐Fe_2_O_3_ at around 35.6°, 62.4°, and 64.0° were attributed to α‐Fe_2_O_3_ according to the standard card (JCPDS 33–0664), corresponding to the (110), (214) and (300) crystal planes, respectively. All the patterns in MXene could be ascribed to typical peaks of Ti_3_C_2_T_x_, which are in agreement with the previous report.^[^ ^31^ ^]^ **Figure** **2**a showcased Raman spectroscopy of the above three samples with chemical information. The Fe−O characteristic peaks proved the existence of α‐Fe_2_O_3_ in M‐Fe_2_O_3_@MXene.^[^ ^32^ ^]^ The band at 564 cm^−1^ was assigned to the defect species related to oxygen vacancies (O_v_) formation in M‐Fe_2_O_3_. In addition, the peaks of LiF/HCl synthesized Ti_3_C_2_T*_x_* at 142 cm^−1^ correspond to the Ti−O vibration of surface groups, peaks at 250 and 600 cm^−1^ represented the out‐of‐plane vibrations (A_1g_) of Ti_3_C_2_ and −OH, and peaks at 400 cm^−1^ represented in‐plane vibrations (E_g_) of Ti−attached function groups atoms (T_x_═O).^[^ ^33^ ^]^ Furthermore, M‐Fe_2_O_3_@MXene's E_g_ and A_1g_ of Ti−O bonding both showed a redshift compared to MXene while the Fe−O characteristic peak presented a blueshift, revealing the change of chemical state of Ti and Fe element,^[^ ^34^ ^]^ which could be also testified by the binding energy of Ti 2p and Fe 2p. Besides, Fourier‐transform infrared spectroscopy (FTIR) of Fe_2_O_3_@MXene was consistent with that of M‐Fe_2_O_3_@MXene, except the adsorption band of M‐Fe_2_O_3_@MXene at 500–600 cm^−1^, which was strongly ascribed to M−O bonds enhanced (Figure S4b, Supporting Information). Thereby, it could be concluded that M‐Fe_2_O_3_ surface successfully facilitates the straightforward self‐assembly and coating of MXene. XPS spectra were conducted to demonstrate the elemental valence to confirm the preparation of precursors and final M‐Fe_2_O_3_@MXene photocatalyst. The presence of Fe, Ti, O, C, N, and F could be inferred (Figure 2b) in characteristic sharp peaks in the XPS spectrum. As illustrated in Figure 2c, the high‐resolution spectrum of Fe 2p of Fe_2_O_3_ before and after KH‐550 modification and M‐Fe_2_O_3_@MXene were almost comparable, exhibiting two spin‐orbit double peaks and two satellite peaks. Among them, the peaks located at 711.7 and 724.8 eV were assigned to Fe^3+^, and the peaks located at 709.9 and 723.0 eV were assigned to Fe^2+^, confirming the presence of O_v_. The shift of Fe 2p XPS peaks toward higher binding energy verified the electron‐deficient properties of Fe species (Figure S5b, Supporting Information).^[^ ^35^ ^]^ Specifically, the O 1s high‐resolution spectrum of Fe_2_O_3_ and M‐Fe_2_O_3_ were fitted to two peaks (Figure S5c, Supporting Information), with the peaks located at 529.3 eV and 530.6 eV attributed to metal‐oxygen bonding (M−O) and hydroxyl oxygen or oxygen vacancies.^[^ ^36^ ^]^ The peaks corresponding to Ti−C, Ti^2+^, Ti^3+^, and Ti−O appearing in the Ti 2p spectra of Ti_3_C_2_ MXene and M‐Fe_2_O_3_@MXene (Figure 2d) agreed well with previous studies.^[^ ^13^, ^37^ ^]^ The weak peak representing the oxidation product TiO_2_ from the M‐Fe_2_O_3_@MXene (pink peaks, 458.8 eV) showed no area enhancement from MXene, indicating an unprevailing effect on the structure and properties. In addition, because of Fe−O−Ti bonding, the low‐valance Ti^3+^ peak became weaker while the Ti^2+^ peak became stronger. Due to the surface chemical interaction, the p‐CO_2_RR performance is greatly enhanced by forming a rapid, higher‐flux charge transfer. The O 1s spectrum could be deconvoluted into four peaks ascribed to the metal oxides and hydroxides (Figure 2e).^[^ ^37b^ ^]^ Additionally, M‐Fe_2_O_3_@MXene displayed a lower binding energy due to the formation of oxygen vacancies and Fe−O−Ti bonding. As for C 1s signals, no significant increase of M−C was observed, suggesting no Fe−C bonding formation (Figure S5a, Supporting Information).^[^ ^14^, ^38^ ^]^ Therefore, the shift in binding energy and Raman spectra signified the establishment of Fe−O−Ti bonding, which was initialized by the electrostatic attraction and oxygen vacancies’ attraction of polar groups on the MXene surface.^[^ ^39^ ^]^ Electron paramagnetic resonance (EPR) is used to detect the oxygen vacancies from unbalanced electron spins in M‐Fe_2_O_3_ and M‐Fe_2_O_3_@MXene samples. As shown in Figure 2f, a Lorentzian EPR single was exhibited in M‐Fe_2_O_3_ at *g* = 2.001, which was an index to an oxygen‐defective structure.^[^ ^36^, ^40^ ^]^ In the case of M‐Fe_2_O_3_@MXene, a similar signal of O_v_ was observed with enhanced intensity, thus indicating more O_v_ are formed with the introduction of titanium species. Meanwhile, light‐irradiated EPR spectra were tested, and the O_v_ signal in M‐Fe_2_O_3_ was slightly increased as active electrons trapped from its CB. Under light irradiation, the peak which is caused by the excited electrons appeared at *g* = 2.006.^[^ ^40^ ^]^ In contrast, the EPR spectra before and after Xe lamp irradiation almost coincide, suggesting efficient migration of photo‐induced electrons in M‐Fe_2_O_3_@MXene. Therefore, this oxygen‐bridged structure played a fundamental role in catalytic CO_2_ reduction regarding the intrinsic reaction activity of electron‐deficient Fe sites as well as charge separation and transfer.

**Figure 3:**
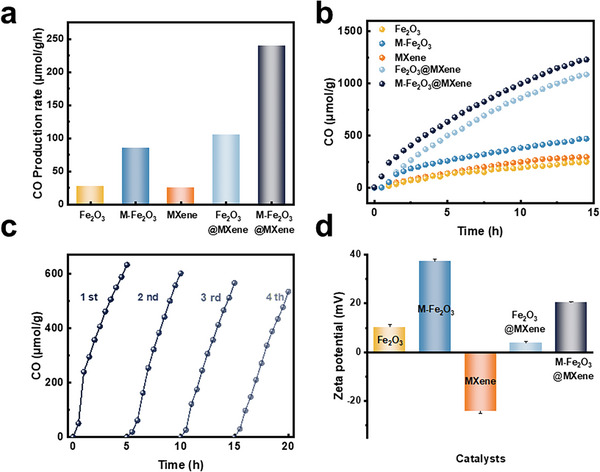
a) Photocatalytic CO_2_ conversion production rate of catalysts. b) Product yield of CO. (c) Photo‐CO_2_RR cycling stability test of M‐Fe_2_O_3_@MXene. and d) zeta potential of catalysts.

To reveal the progressively enhanced photoactivity of Fe_2_O_3_ and M‐Fe_2_O_3_ to the designed M‐Fe_2_O_3_@MXene, the as‐prepared samples were examined for CO_2_RR under simulated sunlight irradiation. From **Figure** **3**a,b, after modifying Fe_2_O_3_ nanocubes by coating a small quantity of −NH_2_, an obvious increase in CO production rate was achieved, testifying to the above‐mentioned conclusions, i.e., modified material has better dispersity in water, exposing more effective interface area with good stability and facilitating the self‐assembly process. Importantly, the photocatalytic CO_2_RR property of M‐Fe_2_O_3_@MXene was expected to be prominently strengthened and showed almost straight‐line growth within 15 h. The maximum reaction rate of CO_2_ conversion over Fe_2_O_3_, M‐Fe_2_O_3_, MXene, Fe_2_O_3_@MXene, and M‐Fe_2_O_3_@MXene catalysts were calculated to be 28, 86, 26, 106, and 240 µmol g^−1^ h^−1^, respectively (Figure 3a). The CO_2_ conversion rate of M‐Fe_2_O_3_@MXene was around 8.6 and 2.8‐fold that of Fe_2_O_3_ and M‐Fe_2_O_3_, respectively. Noteworthily, for the M‐Fe_2_O_3_@MXene catalyst, only CH_4_ evaporation was detected as a by‐product, with a generation rate of 55 µmol g^−1^ during 15 h (Figure S6a, Supporting Information). ^1^H NMR experiments detected no formation of liquid products (Figure S6b, Supporting Information). It could be inferred that the KH‐550 does not participate in the bonding process since Fe_2_O_3_@MXene has similar CO and CH_4_ production rate curves. The inferior stability of MXene‐based catalysts at high temperatures and aqueous environments limits their wider application.^[^ ^41^ ^]^ In the recycling tests of M‐Fe_2_O_3_@MXene, the reaction was restarted every 5 h (Figure 3c). We observed a performance decay in the third recycling, probably caused by the loss of active sites. Even so, the high activity and stability of M‐Fe_2_O_3_@MXene were still maintained. Presumably, the inner nanocubes slow down the deactivation of the MXene membrane. Whereas the reported CO production rate of Fe‐based photocatalysts was at most tens of µmol g^−1^ h^−1^, M‐Fe_2_O_3_@MXene showcased advantageous performance and robust potential in photo‐CO_2_RR with an increase of one magnitude at least. Self‐assembly by static electricity was evident by measuring the Zeta potential at pH = 7 (Figure 3d). Fe_2_O_3_ and M‐Fe_2_O_3_ held a positive charge, while MXene possessed a negative charge, and the resulting composite material Fe_2_O_3_@MXene and M‐Fe_2_O_3_@MXene showed a positive charge. The surface charge state of precursors provided the basis for subsequent chemical bonding. Moreover, the higher zeta potential values of KH‐550‐containing samples indirectly indicate that they were more stable in water with higher dispersion (Figure S7, Supporting Information).^[^ ^42^ ^]^ These results demonstrated the enhanced intrinsic activity of the inner Fe_2_O_3_ nanocubes and accelerated charge flow through an interfacial Fe−O−Ti bond achieved by coating the MXene film.

**Figure 4:**
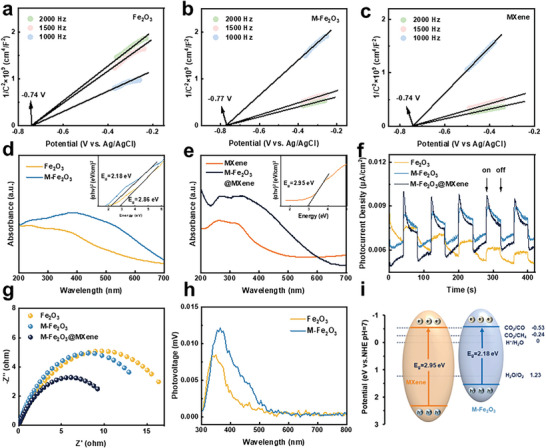
Mott‐Schottky of a) Fe_2_O_3_, b) M‐Fe_2_O_3_, c) MXene. UV–vis diffuse reflectance spectra and band gap of d) Fe_2_O_3_ and M‐Fe_2_O_3_ (inset Tauc plots of Fe_2_O_3_ and M‐Fe_2_O_3_), and e) MXene and M‐Fe_2_O_3_@MXene (inset Tauc plots of MXene and M‐Fe_2_O_3_@MXene). Photoelectrochemical test of photocatalysts, f) photocurrent density, g) EIS tests, h) SPV results of photocatalysts. i) Schematic band structure of M‐Fe_2_O_3_@MXene.

To investigate the mechanism of improved photocatalytic activity of M‐Fe_2_O_3_@MXene, electrochemical tests were carried out to explore the photo‐generated carrier separation and charge transfer. The band edge position of Fe_2_O_3_, M‐Fe_2_O_3_, and MXene was confirmed by electrochemical Mott‐Schottky and UV–vis adsorption plots. **Figure** **4**a–c demonstrates that the conduction band (CB) of Fe_2_O_3_, M‐Fe_2_O_3,_ and MXene was calculated to be ‐0.54, ‐0.57, and ‐0.54 V (vs normal hydrogen electrode, NHE), respectively, demonstrating slightly improved selectivity of CO_2_ reduction to CO. Moreover, their optical absorption edges and band properties were measured by UV–Vis diffuse reflectance spectroscopy. From the Tauc plot (Figure 4d), after modification, it was concluded that the bandgap of Fe_2_O_3_ was narrower, which favored light harvesting. Ultraviolet photoelectron spectroscopy (UPS) studies were conducted to verify the above results. The cutoff energy (*E*_cutoff_) values for M‐Fe_2_O_3_ and MXene were 17.28 and 16.97 eV, and the Femi energy values for M‐Fe_2_O_3_ and MXene were 2.18 and 2.64 eV. Therefore, the work functions (*Φ*) and Fermi‐level (*E*_f_) of M‐Fe_2_O_3_ were 3.94 and ‐0.56 eV for M‐Fe_2_O_3_, and 4.25 and ‐0.25 eV for Ti_3_C_2_ MXene (Figure S8, Supporting Information).

Furthermore, a photocurrent response test was conducted to appraise the separation efficiency of photogenerated carriers under simulated light illumination. Figure 4f revealed that M‐Fe_2_O_3_ performed a much higher transient photocurrent density response compared to Fe_2_O_3_ samples, and M‐Fe_2_O_3_@MXene had the highest photoelectric current density signal, which was evident from electrochemical impedance spectroscopy (EIS) with M‐Fe_2_O_3_@MXene exhibiting smallest resistance in the Nyquist plots (Figure 4g). To further explore the separation efficiency of the photocatalyst, surface photovoltage spectroscopy (SPV) was employed. In Figure 4h, the strong positive signals located at 300–500 nm could be identified as the transfer behavior of holes transferring to the sample surface.^[^ ^43^ ^]^ The positive curvature of Fe_2_O_3_ and M‐Fe_2_O_3_ confirmed their n‐type semiconductor properties with enhanced charge separation efficiency after KH‐550 modification. The above‐mentioned outcomes showed the increased intrinsic activity of Fe_2_O_3_ photocatalyst. Figure 4i shows a schematic of the transfer and separation of photoexcited charge carriers for the self‐assembly of M‐Fe_2_O_3_@MXene nanomaterials and corresponding induced photochemical processes. The staggered band alignment allows for a Z‐scheme mechanism, which was formed by the interfacial chemical bonded Fe─O─Ti. Due to the enhanced interfacial electric field and low charge transfer resistance, an effective M‐Fe_2_O_3_/Ti_3_C_2_ heterojunction accelerates the charge separation, where the photoelectrons in the CB of MXene would transfer to the VB of M‐Fe_2_O_3_. On the surface of M‐Fe_2_O_3_ and Ti_3_C_2_ MXene, p‐CO_2_RR and O_2_ evolution reaction (OER) occurred separately.^[^ ^44^ ^]^

In situ DRIFTS tests of MXene and M‐Fe_2_O_3_@MXene were performed to elucidate the p‐CO_2_RR mechanism. After photoirradiation, a variety of intermediates could be observed in M‐Fe_2_O_3_@MXene, including active CO_2_ molecule (CO_2_*, 1635 cm^−1^), chelating bridged carbonate (c‐CO_3_^2−^, 1673 cm^−1^), monodentate carbonate (m‐CO_3_^2−^, 1528 cm^−1^ and 1507 cm^−1^), bidentate carbonate (b‐CO_3_^2−^, 1393 cm^−1^) and bicarbonate (HCO^3−^, 1720 cm^−1^).^[^ ^45^ ^]^ In addition, the COOH* species showed a distinct signal at 1560 cm^−1^_,_ which was transformed into the absorbed CO (CO*, 2080 cm^−1^). The intensity of the peaks increased with increasing illumination time, indicating that both CO_2_ and H_2_O continuously participated in the photoreduction reaction. Besides, the characterization peak of CH_3_O* (1057 cm^−1^) intermediate was observed, confirming the CH_4_ formation.^[^ ^46^ ^]^ A CH_3_O* peak is also observed with higher intensity in MXene (Figure S9, Supporting Information). However, the other above‐mentioned peaks were rather modest, highlighting the change of active sites for reaction and the efficient activation of CO_2_ together with H_2_O by interfacial bonding.

**Figure 5:**
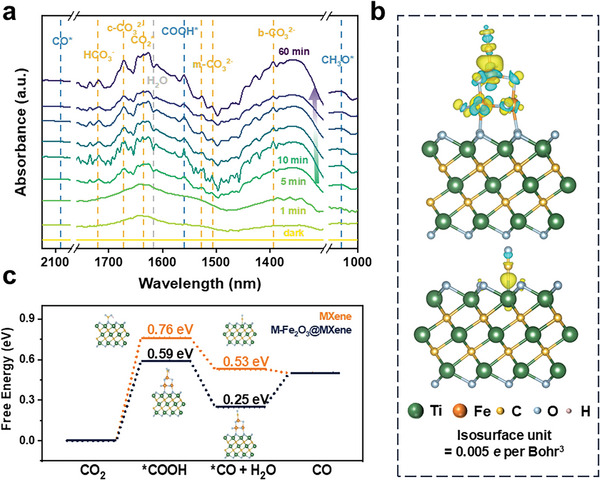
a) In situ DRIFTS spectra of M‐Fe_2_O_3_@MXene. DFT calculations of CO_2_RR on M‐Fe_2_O_3_@MXene and MXene. The charge density difference plot of the *CO adsorption structure for b) M‐Fe_2_O_3_@MXene and MXene. Yellow contours indicate electron accumulation and light green contours denote electron deletion. c) Gibbs free energy profiles for CO_2_RR to CO.

To reveal the reaction mechanism for the enhanced catalytic performance of M‐Fe_2_O_3_@MXene, DFT calculations were conducted according to the atomic structures shown in **Figure** **5**. The model of M‐Fe_2_O_3_@MXene was rationally simplified according to previous research by using a global minimum structure of (Fe_2_O_3_)_2_ cluster connected to the MXene unit and forming a Fe−O−Ti site.^[^ ^47^ ^]^ To explain the charge redistribution at the interface, a differential charge density with *CO adsorption is investigated in Figure 5a,b and Figure S10 (Supporting Information), In M‐Fe_2_O_3_@MXene, a much stronger electronic coupling is observed. It can be inferred that highly delocalized valence electrons (Fe^3+^) play a leading role in influencing the local electron perturbation of the interfacial Fe─O─Ti sites positively.^[^ ^36^, ^48^ ^]^ Due to the tunable electron properties of iron catalysts, the generation of electrons at central Fe sites is beneficial for the separation of photo‐induced carriers, thus initiating a redox reaction with higher intrinsic activity of Fe_2_O_3_. Given the speculation that Fe−O−Ti bonding functions as the active site of the reduction site for photocatalytic CO_2_ reduction, the Gibbs free energy distribution was calculated for each step of CO_2_RR with proposed reaction pathways (CO_2_ → *COOH → *CO → CO), the formation energy barriers of *COOH, the potential rate‐determining step (RDS) for the formation of CO, are significantly reduced by 22% in Figure 5c when comparing the Gibbs free energy changes of M‐Fe_2_O_3_@MXene with MXene.

## 3 Conclusion

In summary, the state‐of‐the‐art CO_2_RR activity for Fe_2_O_3_‐based photocatalyst was achieved by MXene‐tuned hollow Fe_2_O_3_ nanocubes. The highly uniform M‐Fe_2_O_3_@MXene nanocomposites exhibited a CO production rate of 240 µmol g^−1^ h^−1^, outperforming that of the previously reported Fe‐based photocatalysts. The formation of Fe─O─Ti bond and O_v_ significantly increased the intrinsic activity of Fe_2_O_3_ and promoted charge separation and transfer, thus inhibiting the recombination of electron‐hole pairs. By combining Raman, XPS, and DFT calculations results, the active site of M‐Fe_2_O_3_@MXene reduced the energy barrier required for the formation of *COOH intermediate. This study proves an avenue for constructing efficient non‐precious metal‐based nanocomposites for photocatalysis.

## Conflict of Interest

The authors declare no conflict of interest.

## Supporting information



Supporting Information

## Data Availability

The data that support the findings of this study are available from the corresponding author upon reasonable request.
