# Supplementary material for: Boosted Efficiency of Fe2O3 for Photocatalytic CO2 Reduction via Engineering Fe−O−Ti Bonding
Source: Adv Sci (Weinh). 2024 Nov 29;12(3):2409002. doi: 10.1002/advs.202409002 (PMC11744727; doi:10.1002/advs.202409002)
Supplement: Supplementary file 1 — Supporting Information [file ADVS-12-2409002-s001.docx]

Supporting Information

Boosted Efficiency of Fe_2_O_3_ for Photocatalytic CO_2_ Reduction via Engineering Fe−O−Ti Bonding

Jingyi Wu, Wei Wang, Xudan Chen, Qiquan Luo, Changzeng Yan*, Zhen Jiao*, and Yuehui Li*

**Table of Contents**

**Section 1. Experimental Procedures**

**Section 2. Supporting Figures**

**Section 3. References**

**Section 1. Experimental Procedures**

**Chemicals.** K_4_Fe(CN)_6_·3H_2_O, NaOH, PVP (MW:40000, K30), (3-Aminopropyl) triethoxysilane (KH-550), LiF, and acetic acid (C_2_H_4_O_2_) were obtained from Titan Co., Ltd. (Shanghai, China). HCl was purchased from Shanghai Lingfeng Chemical Reagent Co., Ltd. MAX (Ti_3_AlC_2_) was supplied by Jilin 11 Technology Co., Ltd. Deionized water was used throughout the experiments. All experimental chemicals were used directly without further purification.

**Synthesis of Fe_2_O_3_.** Fe_2_O_3_ with cubic structure was synthesized by decomposition and sequential calcination of Prussian blue. Generally, 3.8 g polyvinylpyrrolidone (PVP) and 0.11 g K_4_Fe(CN)_6_·3H_2_O were added to 50 mL HCl solution (0.1 M) under magnetic stirring. After 30 min, the clear solution was heated at 80 °C for 24 h, followed by washing with ethanol and deionized water several times. Fe_4_[Fe(CN)_6_]_3_ (Prussian blue) was obtained by drying at 60 °C in an oven. The solid sample was dispersed into 20 mL of ethanol and was mixed with 3.4 mL of 0.2 M NaOH aqueous solution. The reaction was accomplished by shaking for about 10 min sequentially, and the Fe(OH)_3_ product was collected by several rinse−centrifugation cycles. Hollow Fe_2_O_3_ microboxes were made by annealing the corresponding precursor at 300 °C in air for 6 h.

**Modification of Fe_2_O_3_.** The surface modification of Fe_2_O_3_ was carried out by reflux polycondensation. Specifically, in a three-mouth flask, mix 50 mg of Fe_2_O_3_ powder, 10 mg of 3-triethoxysilane propylamine (KH-550) and 5 mg of carboxylic acid, 5 ml of absolute ethanol and 300 μl of deionized water for 36 hours at 90 °C with a condenser tube and magnetic rod. Afterward, the M-Fe_2_O_3_ is collected by centrifugation, washed with DI water, and dried at 60℃ under vacuum in an oven.

**Synthesis of MXene.** MAX powder (Ti_3_AlC_2_) was used to prepare the MXene with the Ti_3_AlC_2_ formula in a 50 mL Teflon vessel. To selectively etching the Al layers in the MAX phase precursor (Ti_3_AlC_2_), 0.8 g LiF was gradually added to 10 mL HCl under continuous stirring to form HF. Then, 0.5 g of MAX powder was slowly added into the solution pinch by pinch, and the mixture was stirred magnetically at room temperature for 24 h. Afterward, the black acidic mixture was washed with DI water via centrifugation (3500 rpm, 5 min) for multiple cycles until its pH reached 6. The collection of the dark-green, few-layered Ti_3_C_2_T_x_ MXene was carried out by freeze drying under vacuum overnight.

**Synthesis of M**-**Fe_2_O_3_@MXene.** The M-Fe_2_O_3_ samples were collected and added to MXene dispersion, collected by centrifugation, washed with DI water, and dried in an oven at 60 °C under vacuum.

**Synthesis of Fe_2_O_3_@MXene.** The Fe_2_O_3_@MXene samples were generated applying the same method as M-Fe_2_O_3_@MXene, and Fe_2_O_3_ was used instead of M-Fe_2_O_3_.

**Material characterisations.** Field-emission scanning electron microscopy (FESEM, Hitachi Regulus8100), Atomic Force Microscope (AFM, Dimension ICON), and transmission electron microscopy (TEM, FEI, Tecnai G2 F20 and Thermo Scientific, Talos F200X) were used to observe the morphology of as-synthesized materials. The X-ray diffraction (XRD) patterns were recorded by a Rigaku SmartLab diffractometer equipped with Cu Kα radiation (λ=1.5406 Å), and the X-Ray photoelectron spectroscopy (XPS) data was carried out on Thermo Scientific K-Alpha, using monochromatic Al Kα radiation (hγ=1486.6 eV). Band structure was collected by ultraviolet photoelectron spectroscopy (UPS) tests. Raman spectra were recorded by HORIBA FRANCE SAS, Xplora Plus. Electron paramagnetic resonance (EPR) spectra were detected by a EPR spectrometer (Bruker, A300). UV-visible (UV-vis) spectra were collected on Hitachi, U-3900. Surface Photovoltaic Spectroscopy was detected by a Surface Photovoltaic Technique (SPV) setup (CEAULIGHT, CEL-SPS1000). In situ diffuse reflectance infrared Fourier transform spectroscopy (DRIFTS) measurements were carried out on a Nicolet IS50-FTIR spectrometer (Thermo Scientific) equipped with a MCT detector and a designed reaction cell. A thin layer of the sample was loaded on the substrate that is placed in the center of the reaction cell. A flowing CO_2_ gas was introduced to obtain a CO_2_ atmosphere for CO_2_ reduction. At last, the Xe light was turned on and the signal was collected through MCT detector during the reaction.

The photoelectrochemical experiments (Electrochemical impedance spectroscopy (EIS) tests, Mott-Schottky measurements, and photocurrent density-time (i-t) tests were performed by CHI 760E (CH Instruments, Shanghai) electrochemical workstation and standard three-electrode system. An ITO conductive glass substrate (1 cm × 1 cm), evenly coated with as-synthesized catalysts was employed as the working electrode, and Pt plate and KCl-saturated Ag/AgCl electrode were used as the counter and reference electrodes, respectively. The quartz cell was filled with 0.5 M Na_2_SO_4_ electrolyte. The following is the working electrode preparation. 4 mg catalyst powder was dissolved in 1 ml ethanol solution and added with 20 μL 5 wt% Nafion solution, then ultrasonically dispersed for 30 min. Afterward, 10 μL slurry was drop cast onto the ITO glass for drying. Three frequency parameters used in Mott-Schottky measurement are 1000 Hz, 1500 Hz, and 2000 Hz. Electrochemical impedance spectroscopy tests and Photocurrent density-time (i-t) performance were conducted under the irradiation of a 300 W Xenon-lamp at -1.5 V_Ag/AgCl_ and the open-circuit voltage (0.1 V_Ag/AgCl_), respectively.

**Photocatalytic CO_2_ Reduction.** Photocatalytic CO_2_ reduction ability of Fe_2_O_3_, M-Fe_2_O_3_, and M-Fe_2_O_3_@MXene was carried out by gas-solid surface reactions in a 50 mL tailored quartz reaction cell with a Xenon lamp simulating solar light. Before illumination, the reaction system was vacuumed and filled with CO_2_ and CO_2_-saturated water. In a typical measurement, 20 mL of distilled water was added to the bottom of the reactor. Then 0.05 g of photocatalyst was dispersed onto ITO glass (9 cm^2^), supported by a hollow perforated cylinder in the reactor. The reaction was performed with a 300 W Xe lamp as a light source. The reaction system was filled with high-purity CO_2_ gas (80 kPa) for 30 min to reach adsorption-desorption equilibrium before illumination, and the experiment was started upon illumination. During the reaction process, the gas in the reaction cell was detected every 1 h by a GC9700Ⅱ gas chromatograph (GC) equipped with a flame ion detector (GC-FID) and thermal conductivity detector (GC-TCD), and the liquid products were collected and detected by Nuclear Magnetic Resonance Spectroscopy (NMR, WNMR-1400).

**Computational Details.** All density functional theory calculations were performed by the Vienna ab initio simulation package (VASP).^[1]^ The Perdew-Burke-Ernzerhof (PBE) functional was employed to treat the exchange-correlation interactions.^[2]^ To better describe the Fe 3d electrons, The DFT+U approach is used with the values of U=3 eV. The plane-wave basis set with a kinetic energy cutoff of 400 eV, the energy convergence criterion of 10^-4^ eV, the force convergence criterion of 0.02 eV Å^−1^, and a (2×2×1) Monkhorst-Pack k-point sampling was employed for structure relaxation. For different surface models, the bottom layer was fixed. A sufficiently large vacuum gap (>12 Å) was employed to prevent the interaction between neighboring periodic structures. H_2_ and H_2_O were calculated in boxes of 20 Å×20 Å×20 Å with the gamma point only. The free energy diagrams for CO_2_ reduction were calculated referring to the computational hydrogen electrode.^[3]^ The free energy of the gas phase and adsorbed species can be obtained from the following equation:

$$\text{ΔG=}\text{ΔE}_{\text{DFT}}\text{+}\text{ΔE}_{\text{ZPE}}\text{-TΔS}$$

where T was set as 298.15 K. ∆ZPE, and TΔS was the change in the zero-point energy and entropy.

**Section 2. Supporting Figures**

**
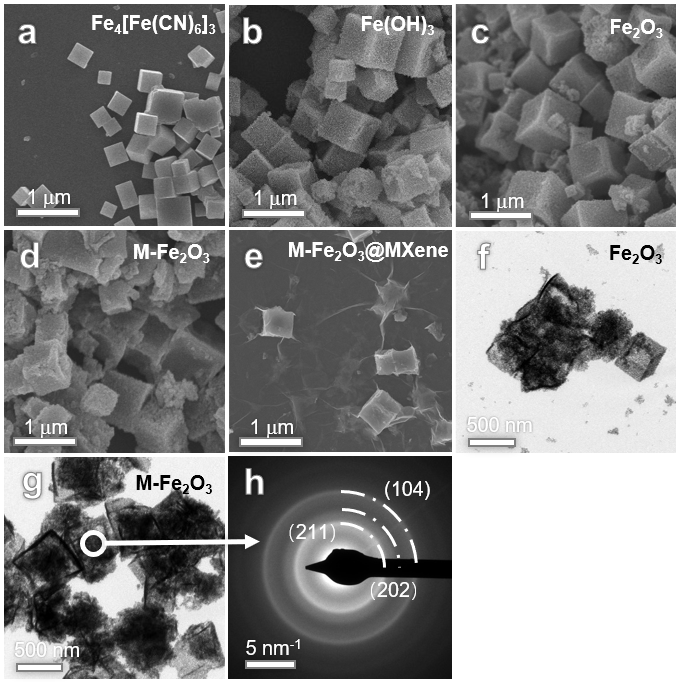
**

**Figure S1.** SEM images of (a) Fe_4_[Fe(CN)_6_]_3_, (b) Fe(OH)_3_, (c) Fe_2_O_3_, (d) M-Fe_2_O_3_, and (e) M-Fe_2_O_3_@MXene, TEM images of (f) Fe_2_O_3_, (g) M-Fe_2_O_3_ and (h) corresponding SAED of M-Fe_2_O_3_.

**
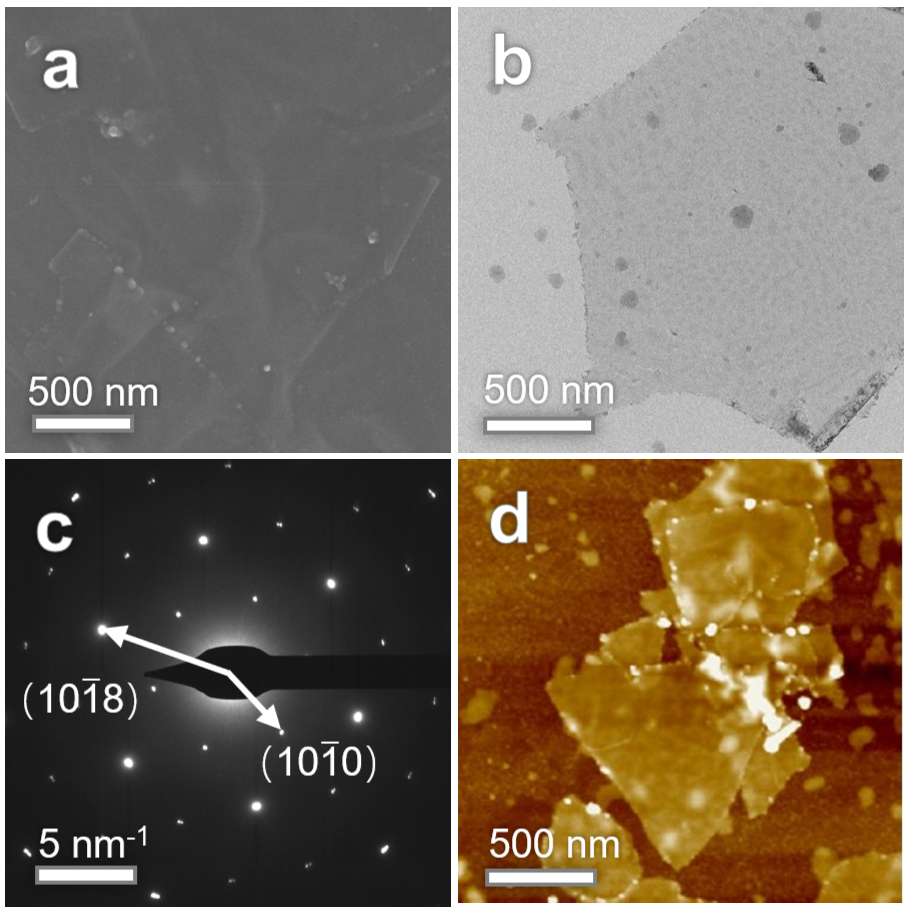
**

**Figure S2. (**a) SEM, (b) TEM images, (c) SAED, and (d) AFM images of MXene.


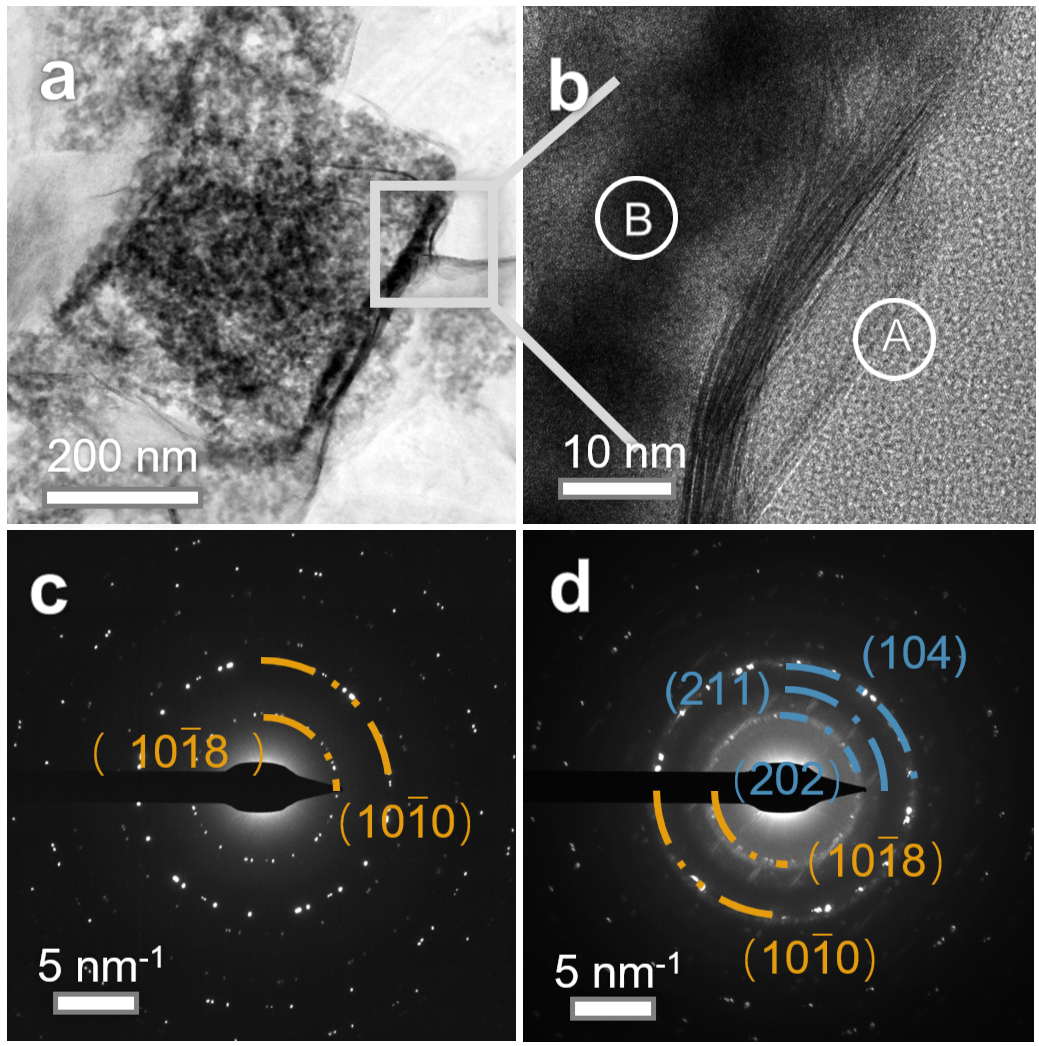


**Figure S3.** (a) TEM images of M-Fe_2_O_3_@MXene. (b) HRTEM images of the contact area of M-Fe_2_O_3_@MXene and corresponding SAED of (c) MXene, region A, and (d) M-Fe_2_O_3_@MXene, region B.


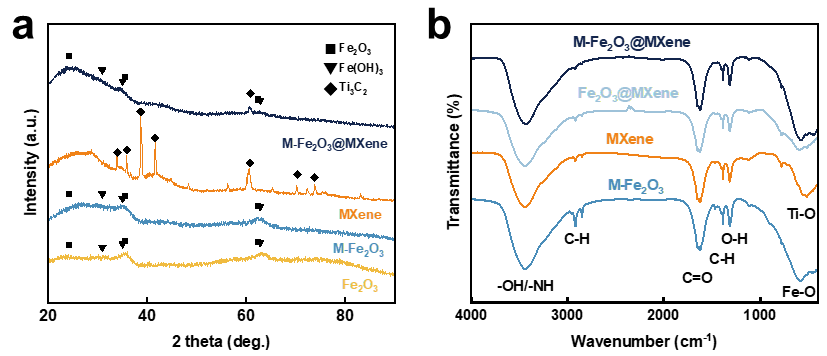


**Figure S4.** (a) XRD results Fe_2_O_3_, M-Fe_2_O_3_, MXene, and M-Fe_2_O_3_@MXene. (b) FTIR results of Fe_2_O_3_, M-Fe_2_O_3_, Fe_2_O_3_@MXene, and M-Fe_2_O_3_@MXene.


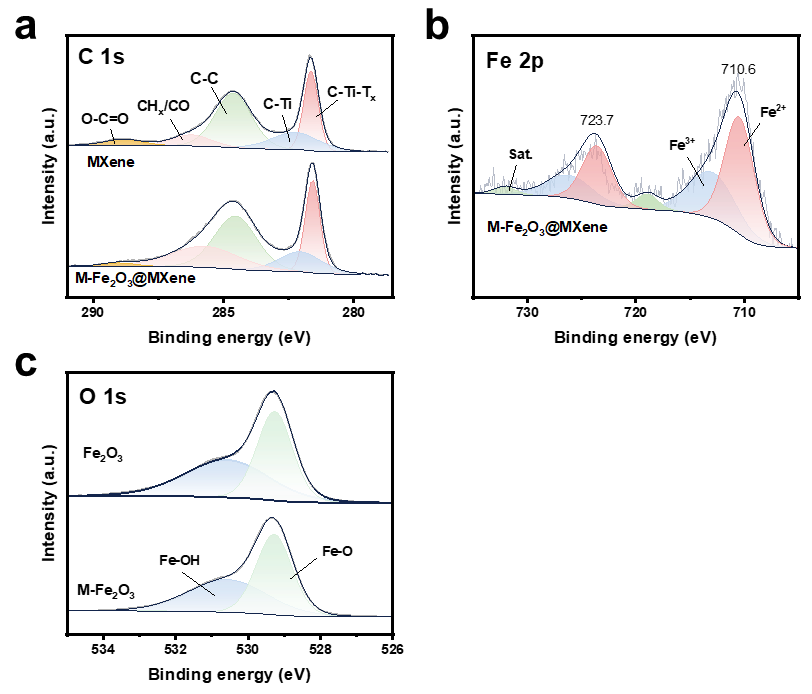


**Figure S5.** XPS study of (a) C 1s spectra of MXene and M-Fe_2_O_3_@MXene, (b) Fe 2p spectra of M-Fe_2_O_3_@MXene, and (c) O 1s spectra of Fe_2_O_3_ and M-Fe_2_O_3_.


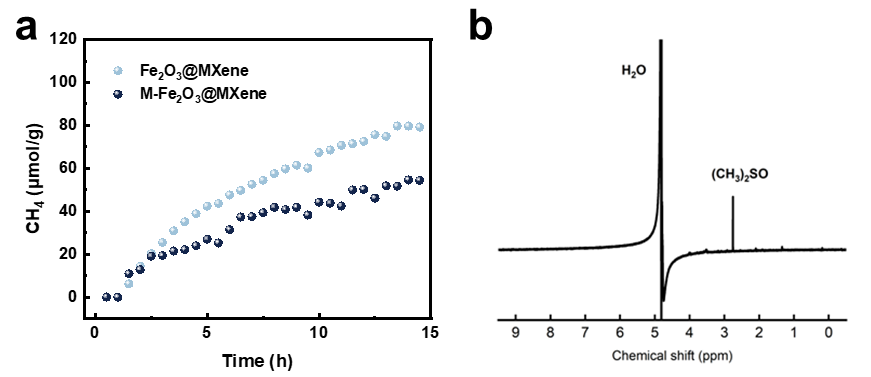


**Figure S6.** Photocatalytic CO_2_ conversion, a) Product yield of CH_4_, b) ^1^H NMR of liquid product.


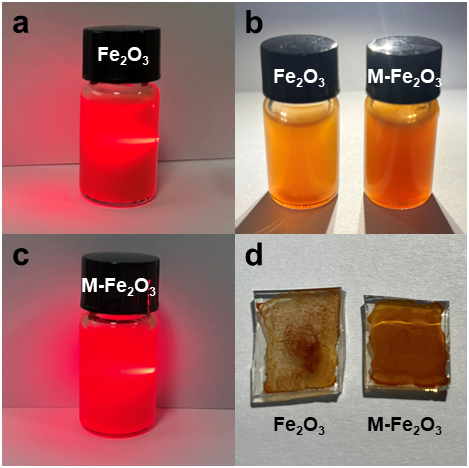


**Figure S7.** The Tyndall effect, in which bottles containing water samples of (a) Fe_2_O_3_ and (c) M-Fe_2_O_3_ are irradiated with a laser pen. (b) images of water samples left for 30 min. (d) samples obtained by drop deposition on ITO.


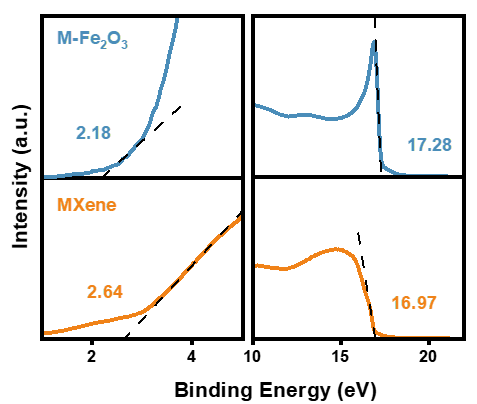


**Figure S8.** UPS spectra of M-Fe_2_O_3_ and MXene.


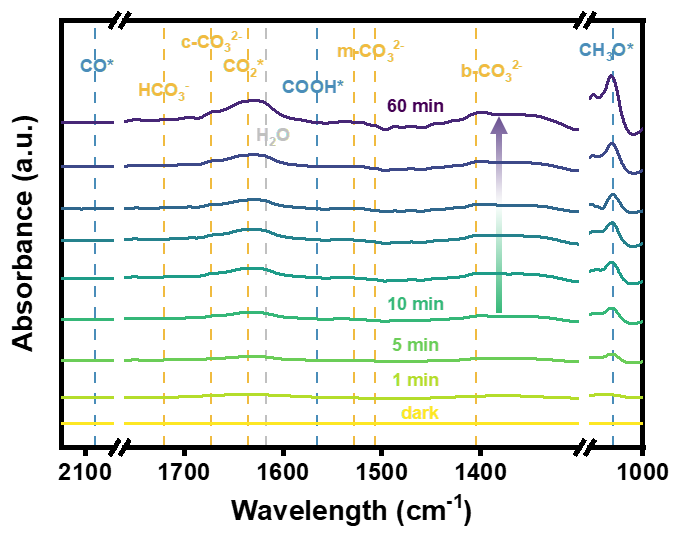


**Figure S9.** In situ DRIFTS spectra of MXene.


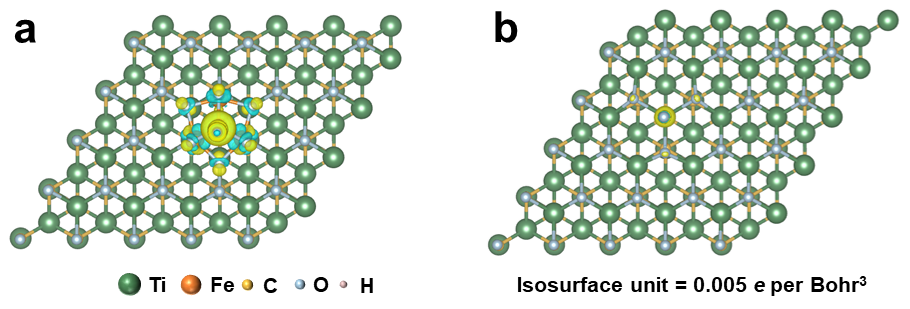


**Figure S10.** The top views of the charge density difference plot of the *CO adsorption structure for (a) M-Fe_2_O_3_@MXene, (b) MXene. Yellow contours indicate electron accumulation and light green contours denote electron deletion.

**Section 4. References**

[1] G. Kresse, J. Furthmüller, *Physical Review B* **1996**, *54*, 11169-11186.

[2] J. P. Perdew, K. Burke, M. Ernzerhof, *Physical Review Letters* **1996**, *77*, 3865-3868.

[3] J. K. Nørskov, J. Rossmeisl, A. Logadottir, L. Lindqvist, J. R. Kitchin, T. Bligaard, H. Jónsson, *The Journal of Physical Chemistry B* **2004**, *108*, 17886-17892.
